# The treatment and survival of patients with triple negative breast cancer in a London population

**DOI:** 10.1186/2193-1801-3-553

**Published:** 2014-09-23

**Authors:** Shrestha Pal, Margreet Lüchtenborg, Elizabeth A Davies, Ruth H Jack

**Affiliations:** King’s College London, Cancer Epidemiology and Population Health, Research Oncology, 3rd Floor, Bermondsey Wing, Guy’s Campus, London, SE1 9RT UK; Public Health England, London Knowledge and Intelligence Team, 2nd Floor, Skipton House, Section C, 80 London Road, London, SE1 6HL UK

**Keywords:** Breast cancer, Triple negative, Treatment, Survival, Population-based

## Abstract

**Purpose:**

Triple negative breast cancer (TNBC) constitutes 10-15% of female breast cancers, and clinical guidelines recommend treatment with chemotherapy and surgery. We examined the recorded treatment and survival of women with TNBC in a population-based sample within the UK.

**Methods:**

Cancer registration data for North East London women diagnosed between 2005 and 2007 were supplemented with pathology data on hormone receptor status to determine triple negative status. Receipt of surgery, chemotherapy, radiotherapy, hormone therapy, or surgery plus chemotherapy according to TNBC status was assessed using logistic regression, and adjusted for age, stage of disease and socioeconomic deprivation. Five-year survival according to TNBC status and treatment was estimated using the Kaplan-Meier method and Cox regression analysis examined adjusted all-cause mortality.

**Results:**

Triple negative status could be determined for 1228 of 2394 women with breast cancer and 128 (10%) had TNBC. Compared to patients without TNBC, patients with TNBC were more likely to receive chemotherapy (fully adjusted odds ratio (OR) =4.21, 95% confidence interval (CI) 2.63-6.75) or surgery plus chemotherapy (fully adjusted OR = 2.52, 95% CI 1.61-3.93). Of patients with TNBC, those who received surgery plus chemotherapy had the greatest 5-year survival estimate (0.74, 95% CI 0.60-0.83). Overall, patients with TNBC had a higher risk of death (fully adjusted hazard ratio (HR) =1.69, 95% CI 1.24-2.30) compared to those without TNBC.

**Conclusions:**

This population-based study found that despite women with TNBC being more likely to receive chemotherapy, or surgery plus chemotherapy, they had a poorer overall survival than with those without TNBC.

## Introduction

Breast cancer is the most common type of cancer diagnosed amongst women in the UK, accounting for 30% of all new cancer cases in women in 2011 (Cancer Research UK). It is increasingly understood as a heterogeneous disease (Metzger-Filho et al. 2012), and one specific subtype of breast cancer that is receiving particular attention is triple negative breast cancer (TNBC). TNBC is defined as a breast cancer that is oestrogen receptor (ER) negative, progesterone receptor (PR) negative and lacks expression of HER-2. This cancer subtype is estimated to account for 10-15% of all breast cancer diagnoses (Dawood 2010), and is associated with poor overall survival (Oakman et al. 2010).

In England, clinical guidelines provided by the National Institute for Health and Clinical Excellence (NICE) recommend surgery and systemic therapy for the treatment of breast cancer (National Institute for Health and Care Excellence 2010). Systemic therapy includes chemotherapy, radiotherapy or hormone therapy, and depends on the tumour hormone receptor status, lymph nodal involvement, and risk of recurrence. Guidelines are not specific to TNBC (National Institute for Health and Care Excellence 2010), and current clinical practice is based upon evidence from clinical trials, which show that chemotherapy combined with surgery is the most effective treatment for patients with TNBC (Isakoff 2010; Rodler et al. 2010; Carey et al. 2010). NICE guidelines recommend adjuvant treatment for early breast cancer with anthracylines and/or taxanes (National Institute for Health and Care Excellence 2010), and encourage the provision of radiotherapy in early or locally invasive breast cancer (National Institute for Health and Care Excellence 2010). Specific guidelines for the role of radiotherapy in TNBC do not exist, so its role is somewhat unclear.

To the best of our knowledge, there have been no population-based studies that have compared a range of treatments received by patients with TNBC, with those for patients without TNBC in the UK. Studies have researched the efficacy of particular treatment options (Isakoff 2010; Rodler et al. 2010; Carey et al. 2010; Colleoni et al. 2010), but none have investigated whether these treatments are being implemented in a London population.

This study follows on from a previous population-based analysis of the hormone receptor status of a cohort of breast cancer patients resident in an area of North East London (Jack et al. 2013). The previous study investigated differences in receptor status amongst women from various ethnic groups, and reported a greater likelihood of TNBC in those from Black and South Asian groups (Jack et al. 2013). Following those findings, this population-based study aimed to compare the treatment received by patients with and without TNBC, to assess their survival, and whether this may be influenced by treatment or other demographic or clinical factors.

## Methods

The occurrence of cancer in the English population is now recorded by the National Cancer Registration Service. During the time period of the study, recording of cancer in the London population was carried out by the former Thames Cancer Registry (TCR). Registrations were initiated by clinical and pathological information received from hospitals and by information about deaths provided by the National Health Service Central Register through the Office for National Statistics. Trained cancer registration officers then extracted further information on demographic details, disease stage and treatment in the first six months after diagnosis from individual medical records. Data were quality assured as they are added to the central database.

Data on 2417 female residents of the area of North East London covered by the former North East London Cancer Network (NELCN) diagnosed with breast cancer (ICD-10 C50) between 2005 and 2007 were extracted from the registration dataset. Breast cancers identified from a death certificate only were excluded (n = 23), leaving a population of 2394 women. For this study, cancer registration data were supplemented with electronic pathology record data received from hospitals in the area. Information on ER, PR and HER-2 receptor statuses was used to derive triple negative disease status. Patients were classified as having TNBC when all three hormone receptor statuses were negative, and as not having TNBC if at least one hormone receptor was present or borderline. Where hormone receptor statuses were a combination of unknown and negative, or all missing, these patients were defined to have an unknown triple negative status.

Information about the type of treatment received within six months (183 days) of diagnosis was identified. The following treatments were studied: surgery, chemotherapy, surgery plus chemotherapy, radiotherapy, and hormone therapy. Age was categorised into 5-year intervals. Due to small numbers in the youngest and oldest age groups, 20–29 and 75 and over age groups were created. As TNM staging data was often incomplete within medical records, TCR used the staging information available to define five categories of stage of disease at diagnosis: 1) localised tumour; 2) extension beyond the organ of origin; 3) regional lymph node involvement; 4) metastatic disease, and 5) not known.

Socioeconomic deprivation was measured by lower layer super output area of residence, based on the income domain of the English Indices of Deprivation 2007 (Communities and Local Government 2007), and grouped into quintiles; 1 (least deprived) to 5 (most deprived). Patients were assigned to a quintile of socioeconomic deprivation based on their postcode of residence.

### Statistical analyses

Logistic regression was used to determine the odds of receiving each type of treatment for patients with and without TNBC, and adjusted for age, stage and socioeconomic deprivation. Five-year survival rates according to TNBC status and treatment were estimated using the Kaplan-Meier method. Cox proportional hazards regression analysis was used to assess all-cause mortality according to TNBC status, adjusted for age, stage of disease, socioeconomic deprivation, and treatment. Survival time was calculated from the date of diagnosis until the date of death from any cause or censored at the end of the follow-up period, on 30^th^ September 2012. Although all data analyses were modelled with the ‘not known’ hormone receptor status group, the main focus is on the comparison between patients with and without TNBC.

## Results

Triple negative disease status could be determined for 1228 of 2394 women diagnosed with breast cancer in the study area of North East London during 2005 to 2007. Of the 1228 women in whom triple negative disease status was determined, 128 (10%) women were diagnosed with TNBC, as previously reported (Jack et al. 2013). This diagnosis was more likely in younger women, those living in more deprived areas, and with a more advanced stage of disease (Jack et al. 2013).

Table 1 shows the numbers and percentages of patients that received each type of treatment within the first six months after diagnosis, as well as the odds ratios (OR) of patients with TNBC receiving each type of treatment compared to patients without TNBC. Adjustment for age, stage and socioeconomic deprivation attenuated the associations somewhat. The likelihood of patients with TNBC receiving surgery was similar to that of patients without TNBC (adjusted OR = 1.05, 95% confidence interval (CI): 0.68-1.62). Patients with TNBC were more likely to receive chemotherapy (adjusted OR = 4.21, 95% CI: 2.63-6.75) and a combination of surgery plus chemotherapy (adjusted OR = 2.52, 95% CI: 1.61-3.93) than patients without TNBC. Those with TNBC appeared to be less likely to receive radiotherapy (adjusted OR = 0.71, 95% CI: 0.42-1.19).

**Table 1:** **Odds ratios (OR) and 95**% **confidence intervals (CI) of receiving each type of treatment comparing female breast cancer patients with TNBC (TNBC), and without TNBC (non-TNBC) (North East London Cancer Network, 2005 – 2007)**

	Number and % of patients receiving treatment									
	TNBC	Non-TNBC			Unadjusted			Adjusted for age, stage and socioeconomic deprivation		
	N	(%)	n	(%)	OR	(95% CI)	*p*	OR	(95% CI)	*p*
*Treatment*										
Surgery	86	(67)	747	(68)	0.97	(0.66, 1.43)	0.869	1.05	(0.68, 1.62)	0.815
Chemotherapy	80	(63)	293	(27)	4.59	(3.13, 6.73)	<0.001	4.21	(2.63, 6.75)	<0.001
Surgery & chemotherapy	53	(41)	208	(19)	3.03	(2.07, 4.44)	<0.001	2.52	(1.61, 3.93)	<0.001
Radiotherapy	20	(16)	249	(23)	0.63	(0.38, 1.04)	0.072	0.71	(0.42, 1.19)	0.194
Hormone therapy	3	(2)	433	(39)	0.04	(0.01, 0.12)	<0.001	0.04	(0.01, 0.12)	<0.001

The 5-year survival rates according to TNBC status and treatment received are shown in Table 2. Patients with TNBC had significantly lower 5-year survival [0.62 (95% CI: 0.53-0.69)] than patients without TNBC [0.75 (95% CI: 0.72-0.78)]. The highest 5-year survival rates were observed for patients without TNBC who received surgery (0.86 95% CI: 0.84-0.89), surgery and chemotherapy (0.86, 95% CI: 0.80-0.90) and radiotherapy (0.87, 95% CI: 0.82-0.91). Among patients with TNBC however, the highest 5-year survival was observed following a combination of surgery and chemotherapy (0.74, 95% CI: 0.60-0.83). Patients with TNBC who received radiotherapy had a much lower 5-year survival of 0.30 (95% CI: 0.12-0.50). Due to the small numbers of patients with TNBC receiving hormone therapy (3) or no treatment (14), these data are not shown.

**Table 2:** **Five-year survival estimates and 95**% **confidence intervals (CI) of patients with TNBC (TNBC) and without TNBC (non-TNBC) - overall survival, and survival by treatment received**

	TNBC		Non-TNBC	
	5-year survival	(95% CI)	5-year survival	(95% CI)
**Overall**	0.62	(0.53, 0.69)	0.75	(0.72, 0.78)
**Treatment received:**				
**Surgery**	0.65	(0.54, 0.74)	0.86	(0.84, 0.89)
**Chemotherapy**	0.65	(0.53, 0.74)	0.77	(0.71, 0.81)
**Surgery plus chemotherapy**	0.74	(0.60, 0.83)	0.86	(0.80, 0.90)
**Radiotherapy**	0.30	(0.12, 0.50)	0.87	(0.82, 0.91)

Hazard ratios (HR) for patients who were diagnosed with TNBC compared with a baseline of patients without TNBC are shown in Table 3. Patients with TNBC had a higher risk of death compared with patients without TNBC (HR = 1.61, 95% CI 1.20-2.17). Adjustments for age, stage of disease and socioeconomic deprivation increased the hazard ratio to 1.84 (95% CI 1.36-2.49). Further adjustment for treatment attenuated this hazard ratio slightly, but it was still significantly higher in patients with TNBC compared with patients without TNBC (HR 1.69, 95% CI 1.24-2.30).

**Table 3:** **Hazard ratios (HR) and 95**% **confidence intervals (CI) and** ***p*** **values according to TNBC status and sequentially adjusted for age, stage, socioeconomic deprivation and treatment**

	HR	95% CI	*p*
Non-TNBC	1.00		
TNBC	1.61	1.20, 2.17	0.002
TNBC (age-adjusted)	2.01	1.49, 2.72	<0.001
TNBC (age, stage, socioeconomic deprivation adjusted)	1.84	1.36, 2.49	<0.001
TNBC (age, stage, socioeconomic deprivation and treatment adjusted)	1.69	1.24, 2.30	0.001

## Discussion

This study of a London population found that women with TNBC were more likely to receive chemotherapy, or a combination of surgery and chemotherapy within six months of diagnosis, compared with patients without TNBC. Five-year survival amongst patients with TNBC was observed to be greatest following surgery and chemotherapy combined, but despite this, patients with TNBC had a poorer overall survival than those without.

Our analysis found that 63% and 41% of patients with TNBC, received chemotherapy and combination therapy, respectively. There are no previous population-based studies in the UK so comparison of the treatment of this population of patients with TNBC with others is difficult. However these findings are consistent with evidence-based treatment recommendations (Isakoff 2010; Rodler et al. 2010; Carey et al. 2010). This study also found that 16% of patients with TNBC received radiotherapy. NICE guidelines for the use of radiotherapy in breast cancer are not specific to TNBC, and state that radiotherapy should be offered in early and locally invasive breast cancer, or where the risk of disease recurrence is high (National Institute for Health and Care Excellence 2010). Therefore treatment with radiotherapy in this cohort may have been determined by the size and spread of triple negative tumours. Interestingly, a recent study has reported that radiotherapy following mastectomy is associated with improved prognosis (Chen et al. 2013), though this has yet to be incorporated into clinical guidelines for TNBC treatment. Patients with TNBC do not usually benefit from hormone therapies such as anti-HER2 or endocrine-directed treatment which are known to be effective in other kinds of breast cancer (Macmillan Cancer Support). Thus, the finding that these patients were much less likely to receive hormone therapy compared to other treatments is likely to reflect good clinical practice.

This observational study found survival was better in patients with TNBC who received chemotherapy, and best following surgery plus chemotherapy, compared with other treatments. This may be partly due to previously reported favourable responses to chemotherapy in TNBC. However, patients with a better prognosis may also have been more likely to have been selected into receiving more aggressive treatment, and this should be taken into consideration when interpreting the survival results. This study also found that the 5-year overall survival rate for patients with TNBC was 62%, compared to 75% in patients without TNBC. This is comparable with several publications which have reported an overall 5-year survival of between 59-77% in patients with triple negative disease (Schwentner et al. 2012; Ovcaricek et al. 2011; Chu et al. 2012; Dent et al. 2007). Our findings also corroborate the previously reported poorer prognosis of patients with TNBC compared with patients without TNBC (Liedtke et al. 2008; Schwentner et al. 2012; Ovcaricek et al. 2011; Wu et al. 2011), even after adjusting for possible confounding factors (Wu et al. 2011; Schwentner et al. 2012; Von Minckwitz & Martin 2012). Of interest are those studies which have reported similar survival of patients with and without TNBC when a complete response of no invasive or in situ residuals in the breast and nodes) can be confirmed by pathology (Schwentner et al. 2012; Wang et al. 2009).

In future studies, it would be important to explore further the treatment and survival of patients with TNBC in other areas of London, and on a national level to investigate treatment patterns and survival across the UK. A larger population of patients with TNBC would also provide more representative results, particularly if more complete data on triple negative status can be obtained. National and local audit of current clinical practice for the treatment offered to and received by patients with TNBC may also represent a profitable area of investigation, as shown previously in Germany (Schwentner et al. 2012). Our study has some limitations. First, a significant proportion of women included in the study had an unknown hormone receptor status due to incomplete pathology data. This may represent the reality of documentation and reporting of disease for large populations in the National Health Service. Ideally, more accurate and improved pathology reporting would enable triple negative disease status to be determined for a greater proportion of patients. Pathology records are now being routinely received by the National Cancer Registration Service and data on triple negative status are now included within registration records. Future studies will therefore be able to make use of larger and more complete samples. Second, treatment data were available for up to six months (183 days) from diagnosis only, which captures the primary treatment received. Again further treatments are now being recorded in registration data, opening up the possibility of studying them and their possible association with survival as well as treatment at recurrence. Third, data on co-morbidities were not included and this may have affected either the treatment that women received, or their prognosis following treatment. Future studies can make use of co-morbidity data now available within cancer registration data from linked Hospital Episode Statistics data on other admissions.

Triple negative disease of the breast is well reported to be an aggressive subtype of breast cancer. This study of a large London population observed that patients with TNBC have poorer overall survival compared to patients without TNBC. However, it is reassuring to find that in line with evidence-based guidelines, women diagnosed with TNBC were more likely to receive chemotherapy or a combination of surgery and chemotherapy, compared to patients without TNBC. This suggests that further population-based studies have potential, along with clinical studies, to contribute to understanding and improving survival for these women.

Funding: This study was carried out by the former Thames Cancer Registry at King’s College London which received funding from the Department of Health. The views expressed in this article are those of the authors and not necessarily those of the Department of Health. This study was completed with the support of the London Knowledge and Intelligence Team, Public Health England.

## Consent

The former cancer registries in England had approval from the National Information Governance Board to carry out surveillance using the data they collected on all cancer patients under Section 251 of the NHS Act 2006. Therefore written informed consent was not required for this study.
